# The Degenerate Infant

**Published:** 1904-02-20

**Authors:** Eric Pritchard


### Hospital Clinics and Medical Progress.

### THE DEGENERATE INFANT.

By Eric Pritchard, M.A., M.D.Oxon., M.R.C.P.Lond.

One of the most obvious and serious effects of over-civilisation in this country is the multiplication of weakly and degenerate children, and since the child is the father of the man this unfortunate condition of affairs must have important bearings on the future of the race. Indeed, according to their various degrees of degeneracy such individuals may become potential or actual sources of danger to society. If we admit that appropriate moral and physical culture can ameliorate the condition of children presenting the lesser degrees of degeneracy and save them from lapsing into serious states of moral delinquency and Hooliganism, it becomes a matter of considerable importance that the diagnosis of degeneracy should be made at the earliest possible opportunity in order that the reforming processes may be set in operation without delay. The stigmata of degeneracy both moral and physical in adults and adolescents are comparatively easy to recognise. In the case of the infant the diagnosis is a more delicate and difficult matter, requiring experience and accurate observation. The more extreme degrees of degeneracy which are characterised by obvious malformations and anatomical deformities, such as hare lip, cleft palate, webbed fingers, congenital tumours, etc., at once attract attention and make the diagnosis plain, but infants presenting the lesser degrees often pass as normal, healthy infants, until at a later date the development of undoubted stigmata reveals our ignorance and disappoints our hopes.

It is therefore to these minor symptoms that I intend chiefly to confine my remarks. As would naturally be expected, it is in the development of the higher nervous functions and of the finer nervous mechanisms that the degenerate organism chiefly fails, and it is therefore to the evolution of the nervous functions that we should most confidently look for early indications of the condition. Among the earlier manifestations of incompetent nervous function must be included the imperfect performance of simple reflex acts in response to normal stimulation; for instance, even before birth, feeble, hesitating fœtal movements are indications that there is something wrong with the nervous mechanism which controls the reflex movements of the embryo; and further the new-born infant who commences his career under conditions which require artificial respiration is less likely to develop into a useful than into an inferior and degenerate member of society. The early performance of the simple automatic or reflex acts, such as those of sucking, of clutching objects with the fingers, and the drawing up of the feet on tickling the soles, are indications of normal development, while their absence or ill co-ordinated performance are signs that the nervous mechanisms which control these acts are functionally inadequate or structurally unsound. Peaceful sleep and active movements during waking hours are further indications of nervous equilibrium and manifestations of normal nerve energy: while sluggish, clumsy, ill co ordinated movements, tremors, spasms, and convulsive muscular contractions are suggestive of a want of nervous co-ordination and stability. A difficulty in training the infant to regular habits such as those of sleeping, feeding, defæcation, micturition, etc., are indications of defective or deficient nervous organisation. These are some of the minor points which distinguish the degenerate from the normal infant. Too much importance must not, however, be attached to the manifestation of individual symptoms; it is only when several symptoms appear in combination that they become diagnostically significant.

I propose now to tabulate a few of the more important stigmata of degeneracy which may be recognised if the various organic systems be carefully examined. Among anatomical stigmata the following are important: gigantism, dwarfism, infantilism, excessive length of the arms, asymmetry of the two sides of the face, head or trunk, asymmetry of the limbs, imperforate anus, long tight foreskin, hermaphroditism, excessive development of hair (especially of soft downy hair on the back and between the shoulders), and albinism.

Among functional stigmata the following must be included: squint, rolling of the eyes, nodding of the head, retraction of the head, convulsions, and epileptiform fits, nervous twitchings, and paralysis. Among disorders of nutrition must be included a tendency to increase very rapidly in weight under normal conditions of alimentation (adiposis) and on the other hand a tendency to leanness under similar circumstances, periodic vomiting or diarrhœa without obvious cause, so-called liver attacks with furred tongue, prominent red papillæ and light-coloured stools, inability to thrive on a properly modified milk diet, idiosyncrasies with regard to special articles of food or to drugs, a tendency to excessive sweating, eczema, spots and rashes of all sorts, red patches or bright coloration with dilated capillaries on the cheeks, precocious intelligence or precocious sexual instincts, late development of the muscular functions, late, irregular, and troublesome teething, a tendency to develop rickets, marasmus, or infantile scurvy on slight provocation, together with feeble resistance to external influences and disease generally. These signs and symptoms, whether singly or in combination, are according to their degree indicative of the degenerate state. From a practical point of view the inability of infants to thrive on an ordinary well-constituted diet is one of the most important indications of a degenerate organisation.

In conformity with the general experience that degenerate individuals tend to respond abnormally or not to respond at all to the ordinary stimuli of their environment, it is found that in the case of infants of this class milk is insufficiently stimulating to induce the secretion of gastric and intestinal ferments which is necessary for its digestion. Consequently such infants must be supplied with food possessing more stimulating properties, such as meat juice or animal extractives, or with prepared foods or peptonised milk, which require little or no further digestion by the ferments of the alimentary canal.

One of the most characteristic features of degenerate individuals generally, and of infants in particular, is that they will not continue to respond for any considerable period of time to monotonous or constant forms of stimulation. They require frequent changes not only of food, but of air, and of the many other elements which constitute the environment. It is the same with all neurasthenics whether old or young, and these varieties of superior degenerates can only be treated with success by complying with these indications. The want of mental and physical powers of application which is almost invariably displayed by degenerates, is explicable when we realize this inherent tendency to tire of constant, similar, and monotonous forms of stimulation. It is half the battle to realize this cardinal fact in the management of degenerate infants. They will not, and do not, thrive on a monotonous diet; even on the very best varieties of natural or modified milk they will become rhachitic, and marasmic, or fall into some other condition of malnutrition which will leave them feeble and delicate individuals for the rest of their lives. I suppose there is no medical man of any experience in the treatment of infants who has not been at times surprised at the manner in which certain infants improve simply by a change of diet, even when this change is contrary to general dietetic principles. The improvement is due to the introduction of a new form of stimulation. How few of us, however, act on this indication, and continue to introduce new changes; we wait for symptoms first, and then try, often most unsuccessfully, to combat them.

In conclusion, I would add that in my opinion one of the most important elements in the successful management of infants is to be able to recognise the signs and symptoms of the lesser degrees of degeneracy, and having recognised them, to conduct the treatment on the principle that the conditions of the environment require frequent change.

